# Complete Regression of Giant Aneurysms in an Infant with Delayed Diagnosis and Refractory Kawasaki Disease via Combination Anticytokine Therapy: Case Report and Review of Similar Cases

**DOI:** 10.1155/2020/6249013

**Published:** 2020-03-28

**Authors:** Maegan Williams, Lakshmi Nagaraju, Mark Gorelik

**Affiliations:** ^1^Division of General Pediatrics, Children's Hospital of San Antonio, Baylor College of Medicine, 333 N Santa Rosa St, San Antonio, TX, USA; ^2^Division of Cardiology, Children's Hospital of San Antonio, Baylor College of Medicine, 333 N Santa Rosa St, San Antonio, TX, USA; ^3^Division of Allergy, Immunology and Rheumatology, Children's Hospital of San Antonio, Baylor College of Medicine, 333 N Santa Rosa St, San Antonio, TX, USA

## Abstract

**Background:**

Kawasaki disease (KD) is an inflammatory vasculitis and is the most common cause of acquired childhood heart disease in developed countries. Current treatment with intravenous immunoglobulin (IVIG) is often ineffective in patients with delayed or refractory disease. We present a case of combination anticytokine therapy in an infant with delayed and refractory KD. *Case Presentation*. A 3-month-old infant presented with refractory KD with giant aneurysms after a delayed diagnosis of one month. Use of combination anticytokine therapy led to resolution of giant aneurysms over approximately 6 months.

**Conclusions:**

Our case is unique in effective use of anticytokine therapy in very delayed disease with giant aneurysms. Additionally, we review other cases for a broader perspective. Prospective study of anticytokine therapy for patients with giant aneurysms may be warranted.

## 1. Background

Kawasaki disease (KD) is an inflammatory vasculitis of medium- and small-sized arteries presenting predominately in children under 5 years old. Diagnosis is clinical and based on criteria including fever, rash, conjunctivitis, changes in the hands and feet, erythema of the oral mucosa and lips, and cervical lymphadenopathy. Incomplete and atypical presentations of KD are also common, often making it a diagnostic challenge [1]. KD is associated with the development of coronary artery aneurysms (CAA) in approximately 20–40% of untreated patients [1]. Early treatment is associated with better outcomes and is anchored by the use of intravenous immunoglobulin (IVIG), but 25% of patients will not have resolution of damage with IVIG. Despite current treatments, KD remains the main cause of acquired childhood heart disease in the US and other developed countries [1]. Those patients who develop persistent CAA despite IVIG treatment are at higher risk of cardiac morbidity not only immediately but also later in life with a mortality rate as high as 5–10% in the 30 years following the diagnosis of KD. The rates of surgical and catheter-based interventions are as high as 50% in those patients with persistent large CAA [2]. Here, we describe a case of a patient with delayed diagnosis of KD and subsequent refractory disease, with complete regression of giant aneurysms after combination anticytokine therapy. We also briefly review similar cases from the literature. Signed parental consent was obtained to present this deidentified case.

## 2. Case Presentation

**Figure 1:**
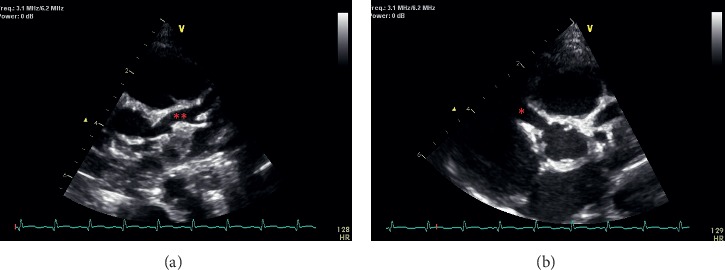
Echocardiogram images from the patient at onset of disease. (a) Parasternal short axis image of the aorta shows a giant fusiform aneurysm of the proximal LAD (^*∗∗*^) and diffuse ectasia of the LMCA. (b) Parasternal short axis image of the aorta shows a giant fusiform aneurysm of the mid RCA (^*∗*^).

**Figure 2:**
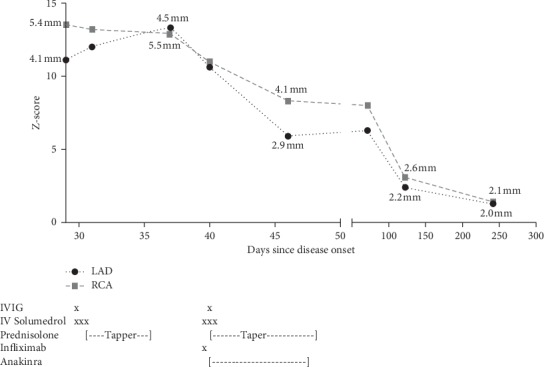
Graphical representation of z-scores over time and interventions administered. LAD = left anterior descending artery; RCA = right coronary artery. *X* represents single dose of medication at specific time point. [---] represents a time period of days the patient received the medication.

A 3-month 25-day-old female, previously healthy, infant presented to the ED with a complaint of 4 weeks of fever, although afebrile for the past 48 hours. On day one of illness, the parents noticed a rash on her feet and the fever started the next day. The rash spread to her legs, chest, arms, and neck. She remained febrile daily and developed congestion and cough. She was seen by her primary pediatrician and was initially thought to have hand-foot-mouth disease. With continued fever, her parents took her to an outside hospital, and she was diagnosed at that time with a URI with reactive airway. She was then treated with amoxicillin and a 5-day course of prednisolone. The steroid treatment cleared the rash, and antibiotics seem to help her congestion per parents. She however continued to have fever and began to have decreased oral intake along with loose stools, conjunctivitis, and peeling of the skin on the feet. She also exhibited increased lethargy and irritability. She presented to our hospital about 1 month into her illness. At time of admission, her last fever was 48 hours prior at 38.6 C. On exam, she was afebrile and had no lymphadenopathy but was noted to have conjunctivitis, systolic murmur II/VI, edema to feet and hands and peeling rash to plantar surface of feet, and mucositis. Admission labs revealed anemia (hemoglobin 7.8 g/dl), thrombocytosis (platelets 917,000 per microliter), elevated ESR (112 mm/hr), elevated CRP (17.26 mg/dL), and hypoalbuminemia (2.4 g/dL). An echocardiogram was obtained showing severe diffuse ectasia of the left main coronary artery and left anterior descending (LAD) and circumflex coronary arteries (Figure 1). The proximal LAD had a fusiform dilation with the largest dimension measuring 4.1 mm (z-score 12.0). There was severe fusiform dilation of the proximal right coronary artery (RCA) measuring 5.4 mm at the largest (z-score 13.0). She was treated with IVIG (2 mg/kg × 1 dose), three days of high-dose “pulse” corticosteroids of 30 mg/kg followed by 2 mg/kg oral prednisolone weaned over two weeks, and low-dose aspirin (5 mg/kg/day). She was started on further anticoagulation therapy with clopidogrel and enoxaparin. She was discharged after 5 days to complete the prednisolone taper. However, 7 days after discharge, she began to have increased irritability and lethargy and developed conjunctivitis and fever up to 38.1 C. Her CRP was again elevated at 6.15 mg/dL, and she was admitted with concern for refractory KD. She had a repeat echo showing persistent large CAA, with some worsening of the CAA size (RCA z-score 13 and LAD z-score 13.3). See Figure 2 for progression of aneurysm size over time. During this second admission, she had a CT angiogram of the chest, abdomen, and pelvis performed showing known CAA of the RCA and LAD but also revealing aneurysms of axillary, brachial, subclavian, intercostal, inferior mesenteric, and left renal arteries. She was treated with a three-day course of high-dose pulse steroids (methylprednisolone 30 mg/kd/day *x* 3), followed by a steroid taper beginning at 2 mg/kg over the next 23 days. She was also treated with anakinra at a dose of 10 mg/kg for 3 days and then 2 mg/kg/day for 11 days for a total of two weeks of treatment. The patient also received her second dose of IVIG of 2 g/kg. She also received one dose of infliximab at 10 mg/kg prior to the anakinra. The patient was able to be discharged from this second admission after 7 days. Fever resolved and did not return. On her subsequent follow-up visits with cardiology, she had serial echocardiogram performed that demonstrated resolution of her CAAs over time. At the 6-month time point after discharge (7 months after disease onset), her coronary artery aneurysms had completely resolved. Furthermore, her multiple other aneurysms had also resolved on repeat CT angiogram performed three and half months after discharge.

## 3. Discussion and Conclusions

Kawasaki disease is an inflammatory condition that typically occurs in children between 6 months and 6 years. The development of coronary artery aneurysms as a sequela of KD can have devastating outcomes for these children, leading to increased morbidity and mortality into adulthood. Current treatment of KD with IVIG is not effective for all patients, and therefore exploration of adjunctive agents for treatment is an active area of investigation [1]. Infliximab is a TNA-*α* antibody which inhibits the action of TNA-*α* both at the receptor level and by removing TNA-*α* from the circulation, and may also inhibit production of other proinflammatory mediators [3]. Recent murine studies suggest that TNF-*α* is a critical factor in generating the generalized myocardial inflammation seen in KD [4]. Anakinra is a recombinant interleukin- (IL-) 1 receptor antagonist. It is used with good efficacy and is very well tolerated in patients with inflammatory diseases such as juvenile idiopathic arthritis and periodic fever syndromes [5]. Recent murine based findings on the role of IL-1 in KD suggest a critical role for this cytokine in the initial inflammatory cascade of KD and thus have prompted clinical trials on the use of anakinra in KD [5]. Therefore, while the etiology of KD still remains an area of investigation, both TNF-*α* and IL-1*β* likely play central roles in this disease, which prompted the targeting of these cytokines in our therapy.

Several reports have been published with remarkable results when anticytokine therapy is used in the treatment of KD. A meta-analysis regarding the effect of infliximab recently published has shown that use of infliximab in IVIG refractory patients has a very strong effect on decreasing the rate of coronary aneurysms with z-scores of >5 [6], while a previous study suggested that infliximab therapy may encourage early regression of aneurysms [7]. While clinical trials of IL-1 blockade are now ongoing [5], only case reports currently exist for use of anakinra for KD. They include the following five cases: The first was a case of a 2-year-old boy who developed KD-associated myocarditis requiring ECMO. He was started on anakinra on day 18 of illness, and his fever disappeared. Three days after his last dose of anakinra, his fever returned along with other symptoms of KD, and echo showed giant CAA on day 53. He was restarted on anakinra on day 54 of illness and continued for 6 weeks. At 6 months, in striking similarity to our case, CAA were no longer evident in this patient [8]. The second case report is an 11-week-old female with KD complicated by macrophage activation syndrome. Treatment with anakinra was started after treatment with IVIG, and corticosteroids resulted in no change in her condition. She also received a single dose of infliximab. After 8 months, she had normalization of mild (z-score ∼2) coronary artery changes on echo [9]. The third case report is of a 3-year-old without CAA from KD but who was resistant to treatment with IVIG. Two weeks after admission, he was started on anakinra and within hours his CRP, ESR, platelets, and hemoglobin normalized [10]. The fourth case reviews an episode of KD in a 16-year-old male patient with aneurysms of between 0.6 and 0.9 cm in all three arteries (approx. z-score 5–11 by our estimated calculations). This patient was treated with IVIG just outside of the 10 day “window” period but had refractory disease as well, and responded with normalization of aneurysms after anakinra for 4 weeks [11]. The final case details response of a child with resistant KD who was then treated with anakinra around one month into disease with significant improvement of z-scores, although not complete normalization. Anakinra treatment in this case was 10 weeks [12]. In contrast to these cases, however a retrospective case series published in January 2018 analyzed multiple cases and found that anakinra showed “neither a striking nor a rapid decrease of coronary dilations” and could not determine if anakinra itself had effects on the CAA [13].

In this report, our patient with refractory and delayed diagnosis of KD, with giant aneurysms of both the right and left coronary arteries (z-score >10) [14], demonstrated complete regression of aneurysms within less than a year of disease onset. The patient's coronary arteries, by size as well as z-score, showed continued regression over time, consistent with a remodeling process. In a study of z-score-defined giant aneurysm regression, only 14% of children showed regression within 1 year, and only 35% after 5 years, making our patient's response atypical [15]. Given this and the other cases described, use of IL-1*β* and TNF-*α* blockade in the form of a combination/high-intensity therapy for KD should be considered for prospective study in patients with giant aneurysms.

One caveat to this case report is that our patient presented with a refractory course which could also be seen in other systemic inflammatory conditions. Patients with polyarteritis nodosa can present with coronary aneurysms [16], and patients with systemic juvenile arthritis have been noted to have coronary changes at the time of diagnosis [17]. Overall, however, due to the presence of mucositis and conjunctivitis, the diagnosis of KD remained the major consideration in this case.

## Abbreviations

KD: Kawasaki disease
CAA: Coronary artery aneurysms
IVIG: Intravenous immunoglobulin
LAD: Left anterior descending artery
RCA: Right coronary artery
IL: Interleukin
TNF-*α*: Tumor necrosis factor alpha.
